# Fever among COVID-19 Patients in a Tertiary Care Hospital of Western Nepal: A Descriptive Cross-sectional Study

**DOI:** 10.31729/jnma.6422

**Published:** 2021-10-31

**Authors:** Prabin Khatri, Aryan Neupane, Ashish Banjade, Ashmita Chhetri, Dipesh Sharma, Pradip Chhetri, Pramila Thapa, Nasatya Khadka, Saugat Karki, Srijana Neupane

**Affiliations:** 1 Department of Internal Medicine, Universal College of Medical Sciences, Bhairahawa, Nepal; 2 Universal College of Medical Sciences, Bhairahawa, Nepal; 3 Karuna Hospital, Budhanilkantha, Kathmandu, Nepal; 4 Department of Community Medicine, Universal College of Medical Sciences, Bhairahawa, Nepal; 5 Civil Service Hospital of Nepal, Kathmandu, Nepal; 6 Kathmandu Valley Hospital, Baghdurbar, Sundhara, Kathmandu, Nepal; 7 Kantipur Dental College and General Hospital, Basundhara, Kathmandu, Nepal

**Keywords:** *COVID-19*, *fever*, *symptoms*

## Abstract

**Introduction:**

COVID-19 has a wide spectrum of clinical presentation ranging from asymptomatic infection to acute respiratory distress syndrome and multi organ dysfunction. Data regarding this is scarce in our setting. This study aims to study the prevalence of fever in confirmed COVID-19 cases in a tertiary care hospital of western Nepal.

**Methods:**

We conducted a descriptive cross-sectional study among patients admitted to COVID-19 wards and intensive care units of a tertiary care hospital. We enrolled patients from August 2020 to January 2021 and the study proposal was approved by the Institutional Review Committee (reference number: 069/20). Convenience sampling method was used. Data entry and descriptive analysis were done in Statistical Package for the Social Sciences version 16.0. Point estimate at 95% Confidence Interval was calculated along with frequency and descriptive statistics.

**Results:**

Among 206 cases of COVID-19, the most common symptom was fever 136 (66.1%) (95% Confidence Interval= 58.14.63-74.05). Sixty-seven (49.3%) of those with fever required intensive care units admission whereas 27 (19.9%) of patients with fever had mortality. Most common comorbidities in the patient having fever is Diabetes mellitus 41 (66.1%) followed by hypertension 20 (62.5%).

**Conclusions:**

Fever was the most common presenting complaint with high prevalence as compared to similar studies done in similar settings. We stress the importance of considering the presence of COVID-19 even in the absence of fever as many patients presented without fever.

## INTRODUCTION

COVID-19 transmission between human to human is due to close contact with the affected, mainly by inhalation of infected respiratory droplets.^1,2^

Nepal reported its first case of COVID-19 in January 2020.^3^ COVID-19 has a wide spectrum of clinical presentations ranging from asymptomatic infection to acute respiratory distress syndrome (ARDS) and multiorgan dysfunction. Fever has been observed as the most common initial presenting complaint in COVID-19 along with cough, sore throat, myalgia, shortness of breath, headache, and loss of smell. Fever ranging from low to high-grade along with or without chills has been found in COVID-19 patients.^4^

Different countries have reported variable clinical presentations and outcomes in patients with COVID-19.^5,6-12^ So, we aimed to find out the prevalence of fever in COVID-19 cases in a tertiary care hospital of western Nepal.

## METHODS

We conducted a descriptive cross-sectional study in patients visiting outpatient departments or admitted to COVID-19 wards and intensive care units of Universal College of Medical Sciences (UCMS). Patients from August 2020 to January 2021 were enrolled after taking ethical approval from the Institutional Review Committee at UCMS (Ref No: 069/20). The patient aged more than 16 years with positive real-time polymerase chain reaction (RT-PCR) assay for SARS-CoV-2 and those who gave written consent were included in this study. Age more than 16yrs who did not give written consent were excluded from the study. Convenience sampling was done and the sample size was calculated as:

n = Z^2^ × p × q / e^2^

  = (1.96)^2^ × 0.5 × (1-0.5) / (0.07)^2^

  = 0.9604 / 0.0049

  = 196

where,

n= required sample size
Z = 1.96 at 95% Confidence Interval (CI)
p = prevalence taken for the maximum sample size, 50%
q = 1-p
e = margin of error, 7%

Adding a 5% non-response rate, the total sample size was 206. Data were entered and analyzed in Statistical Package for the Social Science version Version 16 (SPSS). Point estimate at 95% Confidence Interval and descriptive statistics were calculated.

## RESULTS

Out of the total 206 included cases, 136 (66.1%) (95% Confidence Interval= 58.14.63-74.05) patients had a fever. Among those 136 patients, 86 (63.2%) were male and 50 (36.8%) were female. About 67 (49.3%) of those with fever required ICU admission whereas 69 (50.7%) of those with fever didn't require ICU. Out of 136 who had a fever, 27 (19.9%) of those patients had mortality (Table 1).

**Table 1:** Sex, admission, and mortality in patients with fever (n=136).

Variable	n (%)
**Age Group**	
<50 years	73 (53.7)
>50 years	63 (46.3)
**Sex of the patient**	
Male	86 (63.2)
Female	50 (36.8)
**Admission**	
Non-ICU	69 (50.7)
ICU	67 (49.3)
**Mortality**	
No	109 (80.1)
Yes	27 (19.9)

Among the 136 patients who had fever also had shortness of breath 65 (47.8%), cough 63 (46.3%), myalgia 53 (39.0%), and anosmia 36 (26.5%). Gastrointestinal symptoms in the form of diarrhea were reported by 13 (9.6%) and 3 (1.5%) out of 136 patients with fever (Table 2).

**Table 2:** Frequency of other symptoms in patients with fever (n = 136).

Symptoms	n (%)
Shortness of breath	65 (47.8)
Myalgia	53 (39.0)
Cough	63 (46.3)
Anosmia	36 (26.5)
Headache	14 (10.3)
Sore throat	13 (9.6)
Rhinorrhea	6 (4.4)
Diarrhea	13 (9.6)
Vomiting	2 (1.5)

The frequency of co-morbidities in fever in COVID-19 (Table 3).

**Table 3:** Frequency of comorbidities among those with fever in COVID-19 (n=136).

Variables	n (%)
**Co-morbidities**	
DM	41 (30.1)
HTN	20(14.7)
COPD	4 (2.9)
Asthma	2 (1.5)

The characteristics of those requiring ICU and those not requiring ICU in relation to demographics, presenting complaints, and co-morbidities (Table 4).

**Table 4:** Demographics, Presenting complaints and Comorbidities of patients requiring ICU and those not requiring ICU among patients with fever (n = 136).

Variables	Non-ICU n (%)	ICU n (%)
**Sex**		
Male	67 (51.9)	62 (48.1)
Female	42 (54.5)	35 (45.5)
**Age group**		
<50 Years	91 (73.98)	32 (26.02)
>= 50 Years	18 (21.69)	65 (78.31)
**Presenting Complaints**		
Fever	69 (50.7)	67 (49.3)
Shortness of breath	19 (19.6)	78 (80.4)
Myalgia	44 (62)	27 (38)
Cough	27 (31.8)	58 (68.2)
Anosmia	33 (71.7)	13 (28.2)
Headache	10 (41.7)	14 (58.2)
Sore throat	12 (60)	8 (40)
Rhinorrhea	5 (62.5)	3 (37.5)
Diarrhea	3 (17.6)	14 (82.4)
**Comorbidities**		
DM	16 (25.8)	46 (72.2)
HTN	13 (40.6)	19 (59.4)
COPD	5 (62.5)	3 (37.5)
Asthma	4 (66.7)	2 (33.3)

## DISCUSSION

Our study included 206 RT-PCR confirmed COVID-19 patients. Out of the total population, fever 136 (66.1%) was found to be the most common presenting complaint which is alike to the finding of Guan and colleagues.^6^ In our study 19.9% of those with fever had mortality. A study was done by Chew, et al. also shows the presence of fever as an indicator of adverse outcome in COVID-19.^13^ In infection, fever occurs due to the release of pyrogens, both endogenous and exogenous to the body. These pyrogens cause the release of cytokines namely, interleukin 1, 2, 6 (IL1, IL2, IL6), Tissue Necrosis Factor (TNF), and Interferon-alpha (INF).^14^ The cytokines elevate the hypothalamic set point resulting in elevated temperatures.^15^ In COVID-19, tissue damage or hypoxia causes the release of cytokines thus causing fever.^16^ A study done by Schneider et al. also suggested that fever is an important feature in severe form of covid-19.^17^

Out of the total study population, 59.7% of the patients were <50 years of age and 40.3% were ≥ 50 years of age. This is comparable to a study done by Panthee, et al. which showed an age distribution of 49.4% and 50.6% respectively.^18^ More than half (62.6 %) of the patients were male and 37.4% were female. Similar sex distribution was shown by a study conducted by Gupta, et al.^19^ The reason for male predominance may be because of higher expression of ACE2 receptor in males than that in women.^20^ Furthermore females have a reduced susceptibility to viral infections which could also be due to the protection from X chromosome and sex hormones.^21^ Besides fever, shortness of breath (47.1%), cough (41.3%), and myalgia (34.4%) were the other common symptoms in our study. Bhandari, et al. reported cough (85.71%) to be the most common symptom followed by fever (78.57%), myalgia (64.28%), and dyspnea (28.57%) while Chen, et al. reported shortness of breath to be the 3rd most common presenting symptom (31 %) behind fever (83%) and cough (82%).^5,22^ Shortness of breath was the second most common presenting symptom in our study. This may be because the majority of our sample size is from critical cases requiring ICU admission and fewer patients with milder symptoms seeking medical attention in this region due to fear of acquiring COVID-19 from hospitals. Myalgia during COVID-19 is thought to be due to the effect of proinflammatory cytokines on muscle tissue. TNF-α causes intensified breakdown of muscle proteins and Prostaglandin E_2_(PGE_2_) can increase pain signaling.^23^ Low i.e. 6.8% of the cases were without any symptoms. This is in contrast with the study reported by Mohan, et al. which showed 44.4% of cases showing no symptoms.^24^

This study found that 47.1% of the total cases required admission in COVID-ICU. This is greater than the results shown by Gupta, et al. (16%).^19^ The reason behind this might be the fact that our study center was one of the only two COVID-19 dedicated tertiary centers and also a referral center in our region. We found that 78.31% of cases ≥ 50 years and only 26.02% of patients <50 years required ICU admission. 80.4% of the patients with shortness of breath, 68.2% of patients with cough, 49.3% of patients with fever required admission to ICU. Regarding those with co-morbidities,72.2% of diabetics and 59.4% of hypertensive required ICU admission. A study from India also found that the majority of diabetics with COVID-19 required ICU care.^19^

Out of all cases, mortality was observed in 20.9%. 36.1% of patients ≥50 years and about 10.6% of cases <50 years died. Amongst those with co-morbidities, 32.3% of patients with DM, 21.9% of patients with Hypertension, 37.5% of patients with COPD didn't survive. Gupta, et al. also showed DM to be associated with increased risk of mortality in COVID-19 patients.^19^ A mechanistic review of molecular interaction between diabetes and COVID-19 suggested that higher levels of glucotoxicity, oxidative stress, Renin Angiotensin Aldosterone System (RAAS) alteration, inflammation, Endoplasmic Reticulum stress, and apoptosis in patients with diabetes could explain the increased risk of morbidity and mortality in COVID-19 patients with diabetes.^25^

This study cannot be generalized as it is a single centered study and being a tertiary referral center for COVID-19, most of the sample size was from severe form of cases, therefore creating a possibility of neglect of symptoms from milder forms. Not every symptom available in the literature could be included. Pre hospital use of antipyretics, which is a common practice in our community, may hamper studying the exact grade, duration, and pattern of fever. A larger sample and multicenter study are needed to address these issues.

## CONCLUSIONS

Fever was the most common presenting complaint in our study with high prevalence as compared to similar studiesf done in similar settings. Nearly half of patients with fever required ICU admission. Fever is also common in those patients who had comorbidities. In such cases, other manifestations should be considered. Also we stress the importance of cosidering the presence of COVID-19 even in the absence of fever as many had no fever. We suggest giving emphasis on early diagnosis, identification of comorbid conditions, early isolation, and prompt treatment of all the COVID-19 cases to reduce transmission, morbidity, and mortality.
